# A study of the effectiveness of a detergent-based California mastitis test (CMT), using Ethiopian and Nigerian domestic detergents, for the detection of high somatic cell counts in milk and their reliability compared to the commercial UK CMT

**DOI:** 10.12688/gatesopenres.13369.2

**Published:** 2023-05-17

**Authors:** Jack D. Rust, Michael J. Christian, Ciara J. Vance, Muhammed B. Bolajoko, Johanna T. Wong, Jeimmy Suarez-Martinez, Fiona K. Allan, Andrew R. Peters

**Affiliations:** 1 Bristol Veterinary School, University of Bristol, Langford, Bristol, BS40 5DU, UK; 2 Centre for Supporting Evidence Based Interventions-Livestock, University of Edinburgh, Easter Bush, Midlothian, EH25 9RG, UK; 3 National Veterinary Research Institute, Vom, Nigeria

**Keywords:** Mastitis, somatic cell count, California mastitis test, reagent, sensitivity, specificity

## Abstract

**Background:  **The California mastitis test (CMT) is a simple cow-side indicator of the somatic cell count (SCC) in milk, providing a useful tool in identifying cases of subclinical mastitis in cattle. Mastitis, and in particular subclinical mastitis, is a major concern in Ethiopia and Nigeria, yet detection is challenging due to cost and access to commercial CMT reagents.

**Methods: **Commercially available domestic detergents from Ethiopia and Nigeria were compared (n = 3 for each country) with the UK commercial CMT reagent in their ability to detect high SCC (>400,000 cells/ml milk).  Sensitivity and specificity of the CMT test were calculated for the different detergents and positive and negative predictive values were established.

**Results:  **The average sensitivities of the tests ranged from 28-75% for the Ethiopian detergents and 68-80% for the Nigerian detergents, compared to 76% for the UK domestic detergent.  Test specificities were 84-98%, 93-97% and 96%, respectively.

**Conclusions:** ****Overall, the detergents demonstrated higher specificity than sensitivity. ****Nigerian detergents performed better than the Ethiopian products, however, the study identified suitable domestic detergents from both Ethiopia and Nigeria, comparable to the UK commercial CMT reagent, and we recommend their use as alternative CMT reagents for livestock-keepers to aid in cost-effective diagnosis of mastitis.

## Introduction

Mastitis is regarded globally as the most common infectious disease in dairy cattle, as well as the most economically important ( Halasa *et al.*, 2007; Makolo *et al.*, 2019; Shittu *et al.*, 2012; Suleiman *et al.*, 2013). The disease can be both clinical and subclinical in nature. Clinical mastitis can be visibly diagnosed and treated on routine physical examination, while subclinical mastitis often remains undetected ( Reddy *et al.*, 2014). An array of causative pathogens can be involved, entering the teat canal and multiplying in the udder, however most cases of mastitis are caused by a small group of bacteria including *Staphylococcus aureus*, *Streptococcus uberis*, *Escherichia coli* and *Mycoplasma species* ( Carrillo-Casas & Miranda-Morales, 2012). 

Due to the infectious nature of mastitis-causing pathogens, it is important for the livestock keeper to detect cases of subclinical mastitis in order to maximize cow health and well-being, as well as maintaining herd health ( Kandeel *et al.*, 2018). Subclinical cases of mastitis tend to show no visible changes in udder or milk, however the production of milk reduces and its composition changes, characterised by high somatic cell counts (SCCs) ( Erskine, 2001).

While bacterial culture has traditionally been taken as the gold standard for diagnosis of clinical mastitis ( Dohoo *et al.*, 2011), SCC has also been used as an indicator of mastitis in dairy herds since the 1960s ( Pyörälä, 2003). Somatic cells contribute to natural defence mechanisms and include lymphocytes, macrophages, polymorphonuclear and epithelial cells ( Pillai *et al.*, 2001) and as such, these cells indicate an inflammatory process in intramammary infection. Counting somatic cells is used to distinguish between infected and uninfected quarters ( Carrillo-Casas & Miranda-Morales, 2012), and has long been considered the gold standard in subclinical mastitis screening ( Duarte *et al.*, 2015). The California mastitis test (CMT) is an inexpensive, easily-applicable, cow-side test that enables the subjective assessment of the number of somatic cells present in a milk sample, as an estimation of the probability and severity of intramammary infection ( Schalm & Noorlander, 1957).

The CMT involves combining equal volumes (2–3 ml) of milk and testing reagent, generally the sodium or potassium salts of long chain fatty acids, alkyl sulphates, alkyl sulphonates, alkyl arylsulphonates or alkyl arylsulphates ( Leach *et al.*, 2008). The gentle agitation of milk with these anionic surface-active agents within a CMT paddle causes lysis of somatic cells and the release of cellular DNA. The DNA then agglutinates, giving the sample varying degrees of a ‘slimy’ or mucoid appearance depending on the number of cells within the sample. The extent of the reaction increases with the SCC of the milk ( Leach *et al.*, 2008). The degree of visible agglutination can be subjectively scored based on an ordinal scale and used as a qualitative test to estimate the SCC of milk samples ( Moroni *et al.*, 2018). Sensitivity, specificity and positive predictive values are important test parameters to assess test accuracy, validity and to minimize error, and are calculated at varying thresholds ( Schepers *et al.*, 1997; Thrusfield, 2018a).

In Ethiopia and Nigeria, dairy cattle are essential contributors to the agricultural industry and national economy, yet the productivity and profitability of the dairy subsector remains below potential in both countries ( Abebe *et al.*, 2016; Ameh *et al.*, 1999; Shittu *et al.*, 2012; Tangka *et al.*, 2002). Economic losses to the dairy industry due to mastitis include reduced milk quality and quantity, veterinary costs, culling and deaths ( Degraves & Fetrow, 1993; Seegers *et al.*, 2003). Subclinical mastitis is considered the most damaging and expensive due to the challenge in early detection ( Adamu *et al.*, 2020) although studies on the economic impact of mastitis in both Ethiopia and Nigeria are lacking ( Adamu *et al.*, 2020; Beyene & Tolosa, 2017; FAO, 2014; Mekonnen *et al.*, 2019; Moru *et al.*, 2018).

Commercial CMT reagents are not easily sourced in either Ethiopia or Nigeria, and the cost of importation limits the cost-benefit ratio in identifying and treating mastitis. However, as a domestic detergent-based CMT reagent is accepted in the UK as a cost-effective alternative ( Leach *et al.*, 2008), this project aims to investigate whether domestic detergents available in Ethiopia and Nigeria can also be utilised in the CMT, in place of the UK commercial CMT reagent, to achieve comparable results. This would thereby provide a cost-effective, widely available means of screening for mastitis in both Ethiopia and Nigeria, allowing for targeted treatment and disease control. In turn, this should decrease disease prevalence and production loss, increase herd health and welfare, and maximize milk quantity, quality, and economic value.

## Methods

### Milk samples

The milk samples came from Holstein Friesian cattle, with the majority of samples with high SCC (>400,000) originating from a farm in Medan Vale, Nottinghamshire, based on the known high number of mastitis cases and their willingness to engage with research projects. Other samples were collected from the dairy farm operated by the University of Bristol, based on ease of collection during COVID-19 restrictions. Samples were predominantly collected by veterinarians using aseptic technique (cleaning the teat, wearing gloves and discarding foremilk) ( https://www.livestockdevelopment.co.uk/mastitis.html), with a few (approximately 20) collected by the respective farmers.

Samples were collected between August 2020 and February 2021. All samples collected for the purpose of California mastitis testing were collected in sterile 50 ml universal containers (Teklab) provided by Scarsdale veterinary practice, Derby, were stored at room temperature and tested within 48 hours of collection. All samples collected for the validation study were split into aliquots for SCC analysis and were posted in sterile 30 ml QMMS (Quality Milk Management Services Ltd) sample pots containing a milk preservative (MicroTabs II, Advanced Instruments; standardised across all containers with an 18mg tablet containing 8mg bronopol and 0.3mg natamycin https://www.aicompanies.com/dairy-testing/) within 48 hours of collection. All samples that were tested using the CMT reagents were tested either cow-side or were stored at room temperature, and tested within 48 hours of sample collection with no preservative added. Samples were identified with numbers in chronological order of collection so as to maintain blinding of details such as sample location. Two of the three operators in the validation study were blind to sample location and number as well as CMT reagents used. In order to demonstrate that the technique could be used in LMIC settings, all operators were inexperienced in regards to performing the CMT prior to the study, were given minimal instruction and were trained using an educational video developed for another project ( www.livestockdevelopment.co.uk/mastitis.html Video 11 Californian milk test).

All laboratory SCC results were sent to the respective veterinarians and farmers.

### Dilution study

An accepted UK detergent-based CMT fluid is comprised of 40 ml Fairy liquid detergent (Proctor & Gamble), diluted with 160 ml water, with 1 ml of dark food colouring to enhance visualisation ( Leach *et al.*, 2008). As one cannot assume the domestic detergent available in Ethiopia and Nigeria will have an equal concentration of the anionic surface-active agent required to lyse inflammatory cells in milk, the study first undertook a series dilution test to determine the most effective concentration of each detergent for visualising high SCC (>400,000 cells per ml milk ^1^) and achieving the most comparable results to the UK CMT.

The study used six commercially available domestic detergents from Ethiopia (Reagents 1–3) and Nigeria (Reagents 4–6), and created five different dilutions: 40 ml detergent to 160 ml water; 50 ml detergent to 150 ml water, 60 ml detergent to 140 ml water; 70 ml detergent to 130 ml water; and 80 ml detergent to 120 ml water. Each dilution had the addition of 1 ml of commercially available food colouring from Ethiopia/Nigeria, depending on the detergent country of origin, to enhance visibility.

The five dilutions for each of the six detergents were then tested with 20 quarter milk samples; 20 samples provided clear indication of the most effective dilutions required to indicate subclinical mastitis. All samples were screened for inclusion in the study using the commercial UK CMT to ensure 15 samples with known high SCC values (CMT score 2–3) were included as well as five samples with known low SCC (CMT score 0–1) to test for false positives. Scores were based on reaction descriptions shown in Table 1. 

**Table 1.** Description of the reactions observed in the CMT according to each score category ( Leach *et al*., 2008).

Category	Score	Description of Reaction
Negative	0	Mixture of milk and test fluid remains the same and can easily be shaken.
Weakly Positive	1	Mixture has a slight mucous appearance but can easily be shaken.
Positive	2	Unmistakable mucous formation can be seen, it is possible still to tip a small proportion of the mixture out.
Strongly Positive	3	Jelly-like consistency is formed and it is difficult to shake the mixture. It is no longer possible to tip out any surplus liquid.

### Validation study

To verify that the Ethiopian and Nigerian domestic detergent-based CMT reagents reliably indicate a high SCC (>400,000 somatic cells per ml milk), indicative of mastitis, each reagent was tested with a further 132 quarter milk samples. These samples were screened using the UK CMT upon entry into the study to ensure a range of CMT scores (0, 1, 2 and 3) were present in the validation study.

An additional two operators also tested the 132 samples using each of the six detergent-based CMT reagents, as well as the UK CMT. Both operators were blind to the origin of each reagent so as to reduce bias. Additionally, all samples were submitted for a laboratory SCC reading to confirm the accuracy of the CMT as a diagnostic tool for indicating high SCC samples.

The study identifies the point of significance as 400,000 cells per ml, determined by the laboratory SCC (Fossomatic method, Delta CombiScope – Model FTIR 400, Netherlands), according to the FIL.IDF 148 A: 95 norm. Samples with SCC >400,000 were chosen as the point of significance as a positive indicator of infection and thus we can expect a CMT score of 2–3, and samples <400,000 were deemed as weakly positive to negative, and thus we can expect a score of 0–1 on the CMT.

### Statistical analysis

Validity of the CMT results using different detergents was assessed by comparing test sensitivity and specificity. Sensitivity is the proportion of animals that truly have the disease that test positive, while specificity is the proportion of animals that do not have the disease that test negative. The results of each CMT were validated against a SCC, and sensitivity and specificity were calculated using the formula in Table 2 ( Thrusfield, 2018b). High sensitivity minimises the number of false-negative results, while high specificity minimises the number of false-positive results. Confidence intervals were calculated using Wilson’s method ( Thrusfield, 2018a).

**Table 2.** Calculation of sensitivity and specificity ( Thrusfield 2018a, Thrusfield, 2018b).

Test result	True status	
	Positive	Negative
**Positive**	*a*	*b*
**Negative**	*c*	*d*
**Calculations**		
Sensitivity	*a / (a + c)*	
Specificity	*d / (d + b)*	
Positive predictive value	*a / (a + b)*	
Negative predictive value	*d / (c + d)*	

Positive and negative predictive values were also assessed ( Table 2, Thrusfield, 2018a). These take into account disease prevalence to show the probability of a test detecting a positive, or negative, animal. All calculations were performed in Microsoft Excel (Version 2018).

## Results

### Dilution study

**Figure 1.**
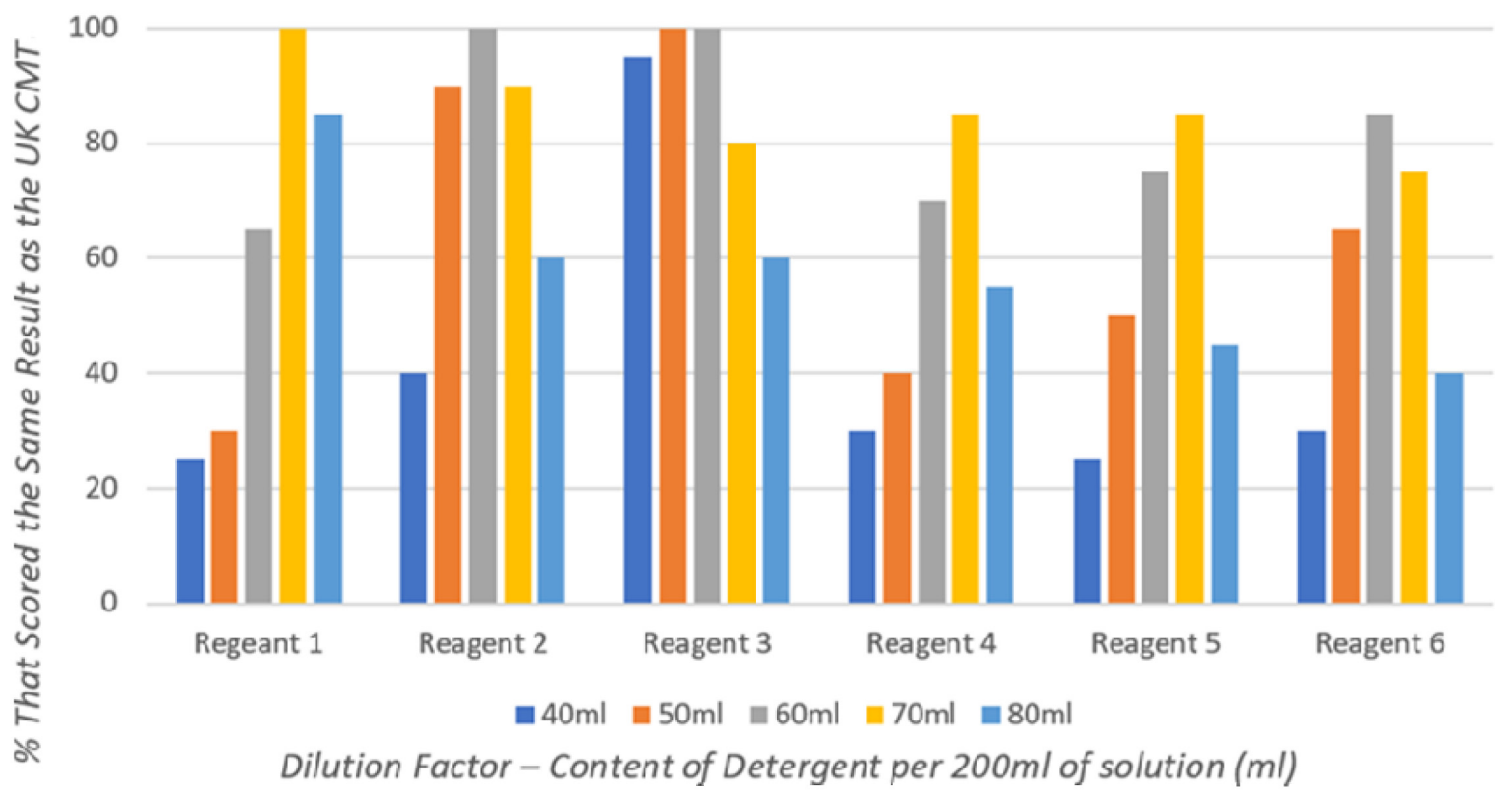
The percentage of detergent-based CMT results that achieved the same result as the commercial UK CMT for each different dilution when testing 20 milk samples.

Individual detergents have differing concentrations of anionic surface-active agent, and therefore, different detergents have varying optimal dilutions to achieve results most comparable to the UK CMT ( Figure 1).

Reagents 1–3 ( Figure 1), made with commercially available Ethiopian detergents, achieved 100% agreement with UK CMT results at varying dilutions: 70 ml detergent per 200 ml solution for Reagent 1; 60 ml per 200 ml for Reagent 2; and both 50 ml and 60 ml per 200 ml, in which this study takes an average of 55 ml per 200 ml, for Reagent 3.

Reagents 4–6 ( Figure 1), made with commercially available Nigerian detergents, are seen to be less efficacious, with none achieving 100% equivalent results to the UK CMT. However, there are clearly optimal dilutions that achieve results most comparable to the UK CMT: 70 ml detergent per 200 ml solution for Reagents 4 and 5; and 60 ml per 200 ml for Reagent 6.

Results obtained from the dilution study are summarised in Table 3, demonstrating the optimum dilution of each detergent selected for the validation study.

**Table 3.** The optimal dilution factor of each detergent to achieve results most comparable to the UK CMT that are then used in the validation study.

Detergent	Country of Origin	Reagent ID	Optimal Dilution
Princess	Ethiopia	Reagent 1 (R1)	70 ml detergent to 130 ml water
Rotana	Ethiopia	Reagent 2 (R2)	60 ml detergent to 140 ml water
Shagan	Ethiopia	Reagent 3 (R3)	55 ml detergent to 145 ml water
Sunlight	Nigeria	Reagent 4 (R4)	70 ml detergent to 130 ml water
Morning Fresh	Nigeria	Reagent 5 (R5)	70 ml detergent to 130 ml water
Mama Lemon	Nigeria	Reagent 6 (R6)	60 ml detergent to 140 ml water

### Validation study

**Figure 2.**
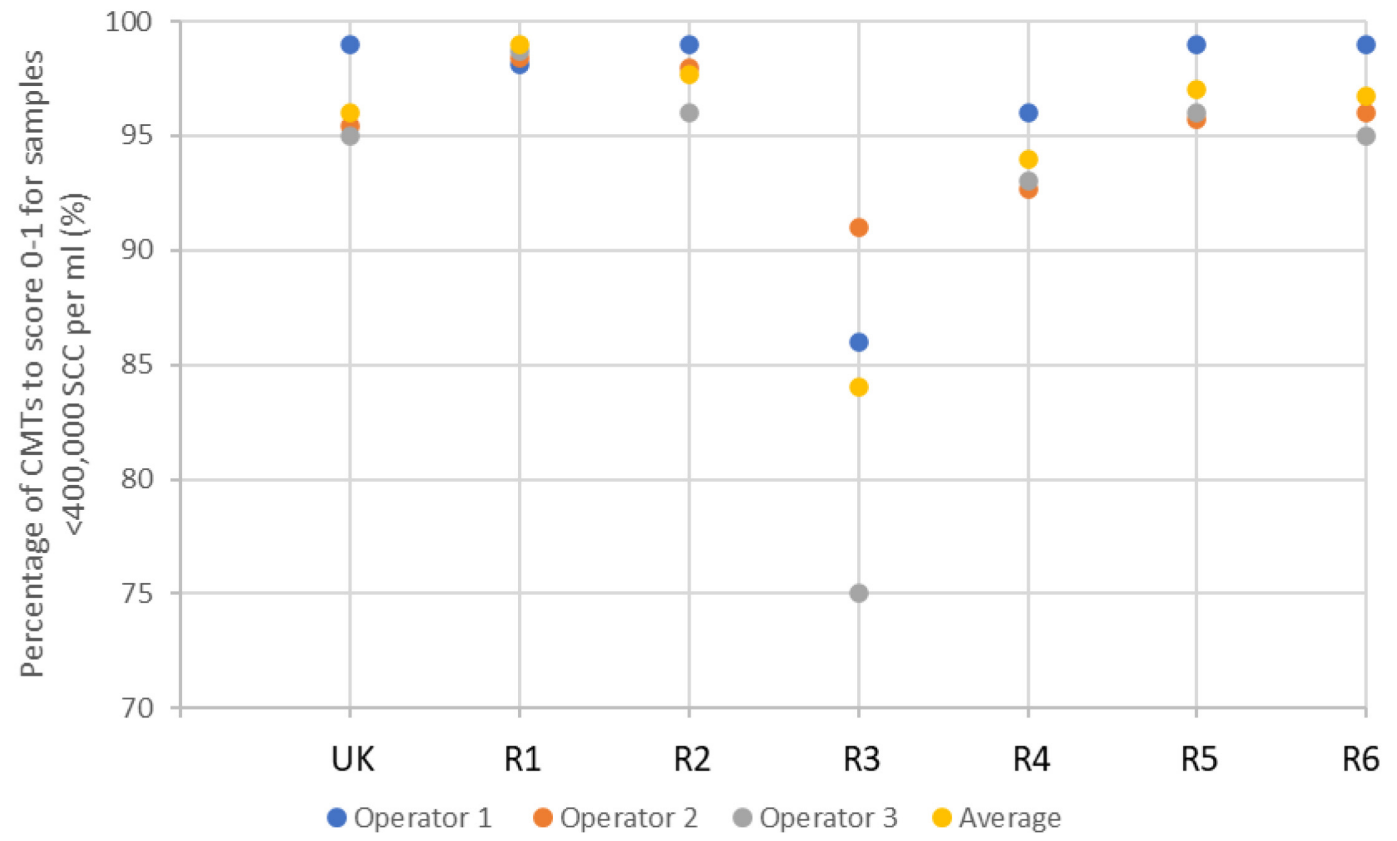
The efficacy of each reagent to score negative to weakly positive CMT results (0–1) across 80 samples, laboratory tested as <400,000 SCC per ml. ‘UK’ indicates UK commercial CMT reagent; ‘R’ indicates reagent.

In the validation study, the majority of reagents scored similarly to the UK CMT, demonstrating greater than 95% specificity ( Figure 2). Reagent 3, however, was a notable exception, scoring lowest, with an average of 84.2% between the three operators. This result, however, is still an acceptable level of specificity.

**Figure 3.**
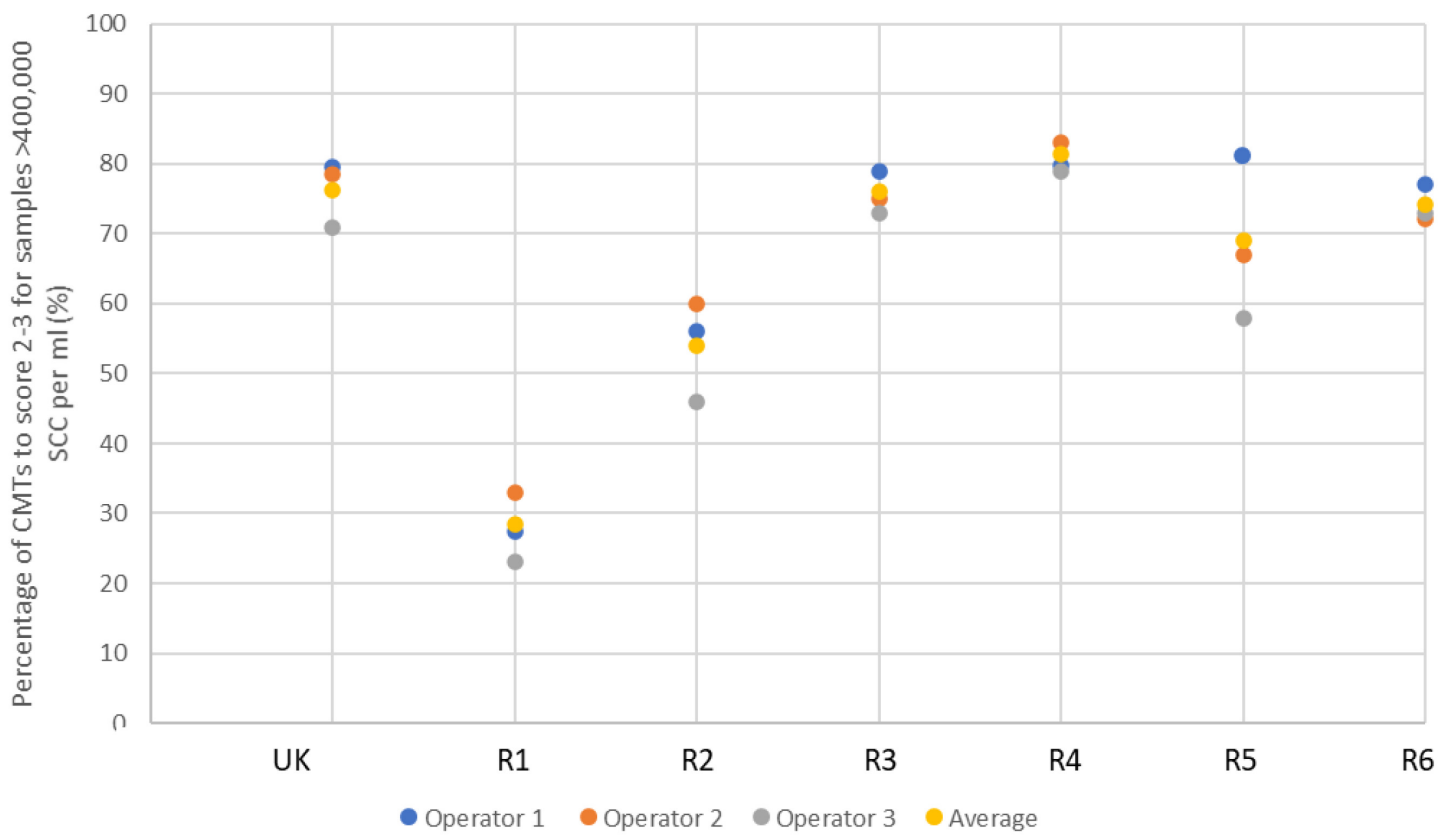
The efficacy of each reagent to score positive to strongly positive CMT results (score 2–3) across 52 samples, laboratory tested as >400,000 SCC per ml. ‘UK’ indicates UK commercial CMT reagent; ‘R’ indicates reagent.

Reagents were tested for sensitivity, by establishing the percentage of high SCC (>400,000) samples each reagent correctly identified as score 2–3 ( Figure 3). It was observed that detergent-based CMT reagents have a higher specificity than sensitivity, and as such are more able to identify low SCC samples. It was, therefore, possible to best differentiate test validity of different detergent-based reagents by their ability to identify high SCC. Reagents 1 and 2 proved to be particularly poor reagents in the identification of samples >400,000 SCC, with 28.3% and 53.3% of samples correctly identified as high SCC, respectively. This is an insufficient proportion of high SCC samples correctly scored, due to the high number of false negative results. With the UK CMT indicating 76.3% of high SCC samples as score 2–3, Reagents 3 and 4 proved to be of similar efficacy, achieving 75.7% and 81.0%, respectively. Reagents 5 and 6 achieved slightly lower sensitivities with 68.7% and 74.3% high SCC samples identified as score 2–3, respectively.

**Figure 4.**
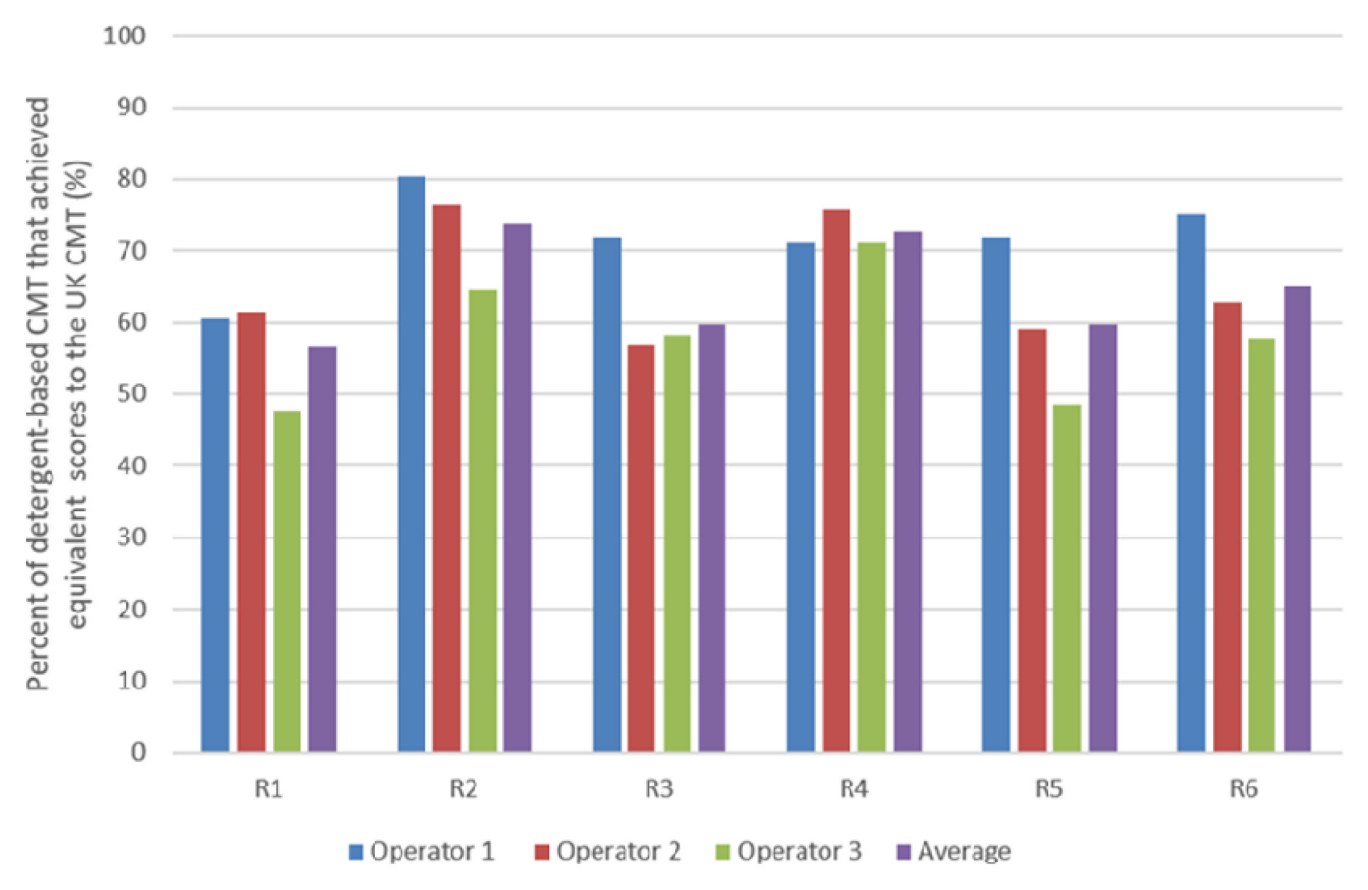
The percentage of detergent-based CMT results that indicated the same SCC (<400, 000 or >400,000) as the commercial UK CMT.

The percentage of detergent-based CMTs that achieved the same interpreted results as the UK CMT, i.e., negative to weakly positive tests score 0–1, and positive to strongly positive tests score 2–3, were established ( Figure 4). Reagent 2 of the Ethiopian detergents (R1-3) is shown to have the highest percentage of similarity to the UK commercial CMT reagent with an average of 74.0% ( Figure 4), despite Reagent 3 demonstrating the greatest sensitivity (of the Ethiopian reagents) ( Figure 3). Of the Nigerian reagents (R4-6), Reagent 4 is shown as achieving the greatest percentage of score similarity to the UK CMT reagent, with an average percentage of 73.3% ( Figure 4).

It can be seen that the UK CMT itself is an imperfect diagnostic tool due to the subjective nature of scoring, achieving an average of 96.3% and 76.3% efficacy at identifying negative to weakly positive, and positive to strongly positive samples, respectively (demonstrated in Figure 2 and Figure 3). Nonetheless, it is accepted for use in the field as an indicator of mastitis and, therefore, alternative detergent-based reagents should be held to similar standards. This suggests, therefore, that a better measure of test validity is achieved through each reagent’s ability to identify high SCC, rather than achieving the same interpreted score as the UK CMT.

 As well as establishing the sensitivity and specificity for each reagent, positive predictive value (PPV) and negative predictive value (NPV) at identifying high SCC (>400,000) samples for each of the detergent-based CMT reagents and the commercial UK CMT reagent were calculated ( Table 4). Almost all reagents demonstrate a high specificity, greater than 95%, with slightly lower specificity for Reagent 4 at 93.8%, and Reagent 3 at 84.2%. Greater differences in sensitivity were observed, ranging from 28.2% for Reagent 1, to 80.8% for Reagent 4. This is reflected in the PPV and NPV, where again Reagent 3 is shown to have the lowest PPV, indicating increased false positives; and Reagents 1 and 2 showing lower NPV, or increased false negatives.

**Table 4.** The average sensitivity, specificity, positive predictive value (PPV) and negative predictive value (NPV) of using the CMT to determine high SCC (>400,000 cells per ml) for different domestic-based CMT reagents across operators. n = 132.

Reagent	Sensitivity		Specificity		PPV	NPV	Range of False Positives	Range of False Negatives
	%	95% CI	%	95% CI				
**UK CMT**	76.30%	63.9 - 86.3%	96.30%	89.6 - 98.7%	93%	86%	1 – 4	11 – 15
**Reagent 1**	28.20%	18.3 - 42.3%	98.80%	93.3 - 99.8%	94%	68%	1 – 1	35 – 40
**Reagent 2**	53.20%	40.5 - 66.7	97.50%	91.3 - 99.3%	93%	76%	1 – 3	21 – 29
**Reagent 3**	75.60%	63.9 - 86.3%	84.20%	74.2 - 90.3%	76%	84%	7 – 20	11 – 14
**Reagent 4**	80.80%	68.1 - 89.2%	93.80%	96.2 - 97.3%	89%	88%	3 – 6	9 – 11
**Reagent 5**	68.60%	55.7 - 80.1%	97.10%	91.3 - 99.3%	94%	83%	1 – 3	10 – 22
**Reagent 6**	74.40%	61.8 - 84.8%	96.70%	89.6 - 98.7%	94%	85%	1 - 4	12 - 14

It would appear overall that Ethiopian detergents are less effective in performing the CMT. However, of the three Ethiopian detergents investigated, Reagent 3 is of highest sensitivity (75.6%, CI% 63.9 - 86.3) and NPV, with the fewest false negatives, and so can be considered the most reliable for detecting mastitis, and thus individual cows requiring treatment, in the Ethiopian setting. In contrast, all Nigerian commercially available domestic detergents (Reagents 4–6), are more efficacious at identifying high SCC milk samples in a detergent-based CMT, with highest sensitivity achieved by Reagent 4 (80.8%, CI% 68.1 – 89.2), which had the fewest false negatives.

## Discussion

The CMT is a common test to indicate infected quarters in cases of subclinical mastitis. Mastitis is a significant disease in both Ethiopia and Nigeria due to its high prevalence and damaging effects on cattle health and productivity. It is, therefore, important for Ethiopian and Nigerian livestock keepers to be able to identify intramammary infections in order to control disease, whether that be to treat, reject the milk, dry off or cull high SCC individuals ( Kandeel *et al.*, 2018).

In the absence of cell counting laboratories, the CMT offers an easy, inexpensive, cow-side test to indicate the presence of infection and subsequent high SCC. However, the cost of reagent importation limits the cost-benefit ratio of the UK commercial CMT. As a domestic detergent-based CMT is accepted in the UK, this study has investigated the use of six detergents, from Ethiopia (Reagents 1–3) and Nigeria (Reagents 4–6) to determine the optimal dilution for use in the CMT, and to validate the sensitivity and specificity these achieve compared to the UK CMT.

All of the Ethiopian detergent-based CMT reagents (R1-3) proved to be less effective than the UK CMT, however Reagent 3 had the highest sensitivity (75.6%, CI% 63.9 - 86.3) and NPV (84%), and therefore, despite the lowest specificity and PPV, was considered the best at identifying disease and identifying cows of likely high SCC to treat. Thus, the authors recommend the use of detergent ‘Shagan’ (Reagent 3), commercially available in Ethiopia, at a dilution of 55ml detergent to 145ml water, with 1ml of indicator, for use in the CMT as an alternative to the UK CMT.

All of the Nigerian detergent-based reagents (R4-6) proved to be more effective than the Ethiopian reagents at identifying high SCC in milk. Reagent 4 proved to have the highest sensitivity (80.8%, CI% 68.1 – 89.2), and NPV (88%), and therefore, the authors recommend the use of the detergent ‘Sunlight’ (Reagent 4), commercially available in Nigeria, at a dilution of 70ml detergent to 130ml water, with 1ml indicator, for use in the CMT to identify high SCC. 

Availability and cost are both important factors in being able to purchase reagents. In selecting detergents for the study, national availability was a selection criterion, along with detergents being in the higher end of the market, as better-quality products had been found to be more effective by Leach *et al.* (2008). For the CMT, the required volume of test fluid per cow is 12ml (3 ml per quarter); one litre of test fluid would therefore be sufficient for 83 cows. At the time of writing, the recommended Ethiopian detergent ‘Shagan’ costs 92–138 Ethiopian Birr (ETB) (equivalent to 2.0–3.1 USD) for one litre. To make one litre of the CMT fluid at the recommended dilution, 275 ml of Shagan is required, equivalent to 25–38 ETB (0.6–0.8 USD), equating to 0.1 ETB per quarter (0.002 USD). The recommended Nigerian detergent ‘Sunlight’ costs 800–866 Nigerian Naira (NGN) (equivalent to 1.9-2.1 USD) for one litre. To make one litre of CMT fluid at the recommended dilution, 350 ml of Sunlight is required, equivalent to 280–303 NGN (0.6-0.7 USD). This equates to around 0.9 NGN per quarter (0.002 USD). Commercial reagent for the UK CMT costs between 6.9–8.3 USD for one litre. The recommended local detergents, therefore, cost around one tenth of the commercial product and are both readily available. To give some context, at the time of the study, the retail price for a litre of milk in Ethiopia ranges from 50-60 ETB (0.9-1.2 USD) and the price for a smallholder selling locally on the informal market is between 30-35 ETB (0.6-0.7 USD) per litre. In Nigeria, these prices range from 250-300 NGN (0.6-0.7 USD) for retail and 150 NGN (0.4 USD) per litre for a smallholder during the rainy season and 250 NGN (0.6 USD) per litre during the dry season.

The study does carry some limitations. Undertaking the study during the COVID-19 pandemic, and ensuring relevant guidelines were adhered to, was an ongoing challenge. In particular, the collection and testing of milk samples proved difficult, with many farms not allowing external access, vet practices frequently only open for emergency cases, and the difficulty in multiple operators testing the samples due to social distancing. This meant the project was very stop-start and took much longer than initially anticipated. Ideally, all samples in the validation study would have been tested by the same three operators, for consistency and to minimise bias, however, due to restrictions this was not possible.

The CMT is subjective in nature, which allows room for bias as well as operator error. A laboratory SCC automatically counts cells within a sample, reducing operator error and removing bias, but it is not without an error margin, assessment of which was beyond the scope of this study. The prolonged retention of samples, postal disturbances, transport and delivery may all contribute to sample modification. Additionally, the SCC also does not take away the value of a clinical exam, as mastitis and other mammary disease may not, in the rare occasion, be reflected in high SCC. All samples in this study are taken from UK Holstein Friesians cows. This study, therefore, assumes that indigenous breeds native to Ethiopia and Nigeria, though managed differently, will respond similarly to the CMT. Pathogens are thought to be similar in African cases, but the availability and types of treatment may be different. Low milk production, how chronic the mastitis is at time of sampling and nutritional status of cows may all effect the sensitivity and specificity when used in the field.

The study identified areas for further research. Firstly, there may have been differences in the efficacy of detergent-based CMT reagents in relation to how long the reagent had been made and left standing; further research is needed to clarify this. Secondly, the Nigerian food colouring was not dark enough and limited visibility, therefore a darker UK food colouring was added to enable testing. Future studies should look at food colouring and indicator dye available in Nigeria for their suitability for use in the CMT. Additionally, to explore the potential for inherent differences in milk from cows raised in different parts of the world, future work could include repeating the work in Ethiopia and Nigeria with different batches of the most effective detergent, in indigenous and exotic breeds of cattle.

## Conclusion

This study supports the use of detergent-based CMTs to improve the health and wellbeing of Ethiopian and Nigerian cattle through the diagnosis and control of mastitis, thereby increasing production and quality of milk, and improving the economic value of the dairy subsector. The most suitable local detergent and dilution was established for both countries and are recommended for use by livestock keepers to aid cost-effective diagnosis of mastitis and importantly reduce herd-level prevalence. This research is contributing to a larger ongoing study into mastitis in both Ethiopia and Nigeria, and will be used to produce educational pamphlets and videos as part of outreach activities to further the promotion of the CMT for the control and management of mastitis.

## Data Availability

Harvard Dataverse: Replication Data for: A study of the effectiveness of a detergent-based California mastitis test (CMT), using Ethiopian and Nigerian domestic detergents, for the detection of high somatic cell counts in milk and their reliability compared to the commercial UK CMT. https://doi.org/10.7910/DVN/PULPBI ( Vance, 2021). This project contains the following underlying data: Dilution_Study_RAW DATA.tab Dilution_Study_Statistical_Analysis.tab Data are available under the terms of the Creative Commons Zero "No rights reserved" data waiver (CC0 1.0 Public domain dedication).
