# Supplementary material for: A study of the effectiveness of a detergent-based California mastitis test (CMT), using Ethiopian and Nigerian domestic detergents, for the detection of high somatic cell counts in milk and their reliability compared to the commercial UK CMT
Source: Gates Open Res. 2023 May 17;5:146. Originally published 2021 Sep 20. [Version 2] doi: 10.12688/gatesopenres.13369.2 (PMC10285044; doi:10.12688/gatesopenres.13369.2)
Supplement: Peer review history

# Peer review: Reviewer response for version 2

**DOI:** 10.21956/gatesopenres.15988.r33356

**Published:** 2023-06-06

**Authors:** Katharine A. Leach

**Affiliations:** 1 Quality Milk Management Services, Easton Hill, UK

The revisions have improved clarity, thank you but a few more improvements could be made.

 It would be helpful to end the introduction by stating:

 A project was undertaken to determine i) appropriate dilutions for African domestic detergents, using a commercial CMT fluid as Gold Standard (Dilution Study)

 ii) the relative performance of a number of African detergents, using laboratory SCC as Gold Standard (Validation Study).

 It is still not entirely clear how samples were dealt with. Is it the case that:

 Duplicate samples were taken from individual quarters of individual cows – one into a sterile 50 ml “Universal” sample tube and one into a clean 30 ml pot containing a milk preservative. A total of XXX samples were taken overall. All California Mastitis Tests were carried out on the samples without preservative, within 48 hours, after storage at room temperature. All SCC tests were carried out on the samples containing preservative; these were posted to a laboratory within 48 hours of collection.

 For the dilution study, only a CMT was needed, therefore a single sample was taken (or used).

 The section on operators really needs another sub-heading.

 For clarity, in the methods could you state that percentage agreement with UK CMT is reported for both studies, and operating characteristics against a Gold Standard of SCC was evaluated in the validation study. And be sure to make clear the comparator throughout each section. It is still not immediately clear **all the way through** with what the individual detergents are being compared at each stage.

 I would still like to see descriptive statistics describing the range and distribution of SCC values for the samples included in the validation test, expecting to see a good spread to give confidence in  the robustness of the evaluation, and a context for the specificity and sensitivity, which will be influenced by the prevalence of disease.

 Overall a useful exercise and encouragement that an affordable cowside test can be provided in Africa. The African costs are useful to see.

 Please check the English in the final paragraph.

Is the work clearly and accurately presented and does it cite the current literature?

Partly

If applicable, is the statistical analysis and its interpretation appropriate?

Partly

Are all the source data underlying the results available to ensure full reproducibility?

Yes

Is the study design appropriate and is the work technically sound?

Yes

Are the conclusions drawn adequately supported by the results?

Yes

Are sufficient details of methods and analysis provided to allow replication by others?

No

Reviewer Expertise:

Dairy herd health; mastitis diagnostics

I confirm that I have read this submission and believe that I have an appropriate level of expertise to confirm that it is of an acceptable scientific standard.

# Peer review: Reviewer response for version 2

**DOI:** 10.21956/gatesopenres.15988.r33355

**Published:** 2023-06-01

**Authors:** John Fishwick

**Affiliations:** 1 Royal Veterinary College, London, UK

I am satisfied with the changes and am happy to recommend publication

Is the work clearly and accurately presented and does it cite the current literature?

Yes

If applicable, is the statistical analysis and its interpretation appropriate?

Yes

Are all the source data underlying the results available to ensure full reproducibility?

No source data required

Is the study design appropriate and is the work technically sound?

Partly

Are the conclusions drawn adequately supported by the results?

Yes

Are sufficient details of methods and analysis provided to allow replication by others?

Partly

Reviewer Expertise:

Pestiviruses and infectious diseases in cattle. I am not research active at present and my background is predominantly clinical work and teaching/course adminstration.

I confirm that I have read this submission and believe that I have an appropriate level of expertise to confirm that it is of an acceptable scientific standard.

# Peer review: Reviewer response for version 1

**DOI:** 10.21956/gatesopenres.14614.r32872

**Published:** 2023-02-13

**Authors:** John Fishwick

**Affiliations:** 1 Royal Veterinary College, London, UK

This is a useful article. Mastitis is a major cause of disease poor welfare and economic loss in cattle. The ability to detect it, especially in sub-clinical stages cheaply and reliably can have an impact on food security and animal welfare throughout the world.

 The paper regularly refers to laboratory SCC test as a comparison for the tests being used here - but it is not stated what the laboratory test is and this is a problem as every test will have some limitations.

 The CMT is usually run as a "cowside" test immediately after the milk is stripped from the cow. In this study the milk has preservative added. It is not stated what the preservative is or how much is added.

 There is no mention of storage conditions or refrigeration of the samples. Samples are then posted within 48 hours. These are all variables that need to be taken into account. For example some cow side testing with fresh milk and no preservative could have been included to the study to see if comparable results were obtained to samples handled in the way described here.

 There is the possibility of variation between batches of the commercial detergent and there is no suggestion as to whether results from different batches were compared. This would have made the results more reliable. Were the results quoted in the paper all from the same batch of detergent or were several different batches used?

 If the test reagents are to be used in Nigeria and Ethiopia it would be helpful, after this UK based data had been obtained, to see if comparable results were obtained in these countries. There may be inherent and unknown differences in the milk from cows raised in a different part of the world which might give rise to different results. Its unlikely but it would be useful to test this.

 More detail could have been given about how the results were scored - perhaps some illustrations would be helpful as the scoring is always going to be quite subjective.

 I think it would have been sensible for the authors to at least comment on some of these factors in the discussion. These points ideally should be included or acknowledged by the authors.  

 Having said all this I feel the paper would be of benefit and value and could help to improve welfare and productivity.

Is the work clearly and accurately presented and does it cite the current literature?

Yes

If applicable, is the statistical analysis and its interpretation appropriate?

Yes

Are all the source data underlying the results available to ensure full reproducibility?

No source data required

Is the study design appropriate and is the work technically sound?

Partly

Are the conclusions drawn adequately supported by the results?

Yes

Are sufficient details of methods and analysis provided to allow replication by others?

Partly

Reviewer Expertise:

Pestiviruses and infectious diseases in cattle. I am not research active at present and my background is predominantly clinical work and teaching/course adminstration.

I confirm that I have read this submission and believe that I have an appropriate level of expertise to confirm that it is of an acceptable scientific standard, however I have significant reservations, as outlined above.

# Response

**Published:** 2023-03-13

**Authors:** Andrew Peters

Dear Reviewer,

 We sincerely thank you for your time and effort in providing expert assessment of our manuscript. There are constructive points raised which we have considered and hope that we have addressed suitably.

 Response to comments are listed in italics font below, with proposed additions and amendments highlighted in bold.

 This is a useful article. Mastitis is a major cause of disease poor welfare and economic loss in cattle. The ability to detect it, especially in sub-clinical stages cheaply and reliably can have an impact on food security and animal welfare throughout the world.

 1. The paper regularly refers to laboratory SCC test as a comparison for the tests being used here - but it is not stated what the laboratory test is and this is a problem as every test will have some limitations.

*The SCC was determined using the **Fossomatic method (Delta CombiScope – Model FTIR 400, The Netherlands), according to the FIL .IDF 148 A: 95 norm.***

*The test counts and approximates the number of somatic cells per ml of milk.*

*2. *The CMT is usually run as a "cowside" test immediately after the milk is stripped from the cow. In this study the milk has preservative added. It is not stated what the preservative is or how much is added.

*Preservatives were only added to the samples that were sent off for a SCC reading – not a cowside test. The preservative was Broad Spectrum **MicroTabs II from Advanced Instruments** - they contain bronopol and natamycin.*

***All samples that were tested using the CMT reagents were tested either cowside or within 48 hours of sample collection with no preservative added**.*

*3.  *There is no mention of storage conditions or refrigeration of the samples. Samples are then posted within 48 hours. These are all variables that need to be taken into account. For example some cow side testing with fresh milk and no preservative could have been included to the study to see if comparable results were obtained to samples handled in the way described here.

*Only samples for SCC testing contained preservatives. They were posted within 48 hours. Samples were not refrigerated but kept at room temperature, as the lab requested.*

***Samples that were tested using the CMT reagents were stored at room temperature, and tested within 48 hours of collection with no preservative added.***

*In the manuscript we state that ’All samples collected for the purpose of California Mastitis Testing were collected in sterile 50 ml sample pots provided by Scarsdale veterinary practice, Derby, were stored at room temperature and tested within 48 hours of collection.  All samples collected for the purpose of somatic cell count analysis were collected in sterile 30 ml QMMS sample pots containing a milk preservative and posted within 48 hours of collection.’  We will add **All samples that were tested using the CMT reagents were tested either cowside or within 48 hours of sample collection with no preservative added**.*

*4.  *There is the possibility of variation between batches of the commercial detergent and there is no suggestion as to whether results from different batches were compared. This would have made the results more reliable. Were the results quoted in the paper all from the same batch of detergent or were several different batches used?

*This study was carried out on UK milk samples with a single batch of each detergent.*

*5.  *If the test reagents are to be used in Nigeria and Ethiopia it would be helpful, after this UK based data had been obtained, to see if comparable results were obtained in these countries. There may be inherent and unknown differences in the milk from cows raised in a different part of the world which might give rise to different results. Its unlikely but it would be useful to test this.

***To explore the potential for inherent or unknown differences in milk from cows raised in different parts of the world, future work could include repeating the work in Nigeria and Ethiopia with different batches of the most effective detergent in local and exotic breeds of cattle.***

*6.  *More detail could have been given about how the results were scored - perhaps some illustrations would be helpful as the scoring is always going to be quite subjective.

***In order to demonstrate that the technique could be used in LMIC, novice operators were used.  They were given minimal instruction and were trained using a video developed for another project ( www.livestockdevelopment.co.uk/mastitis.html Video 11 Californian Milk Test).***

# Peer review: Reviewer response for version 1

**DOI:** 10.21956/gatesopenres.14614.r31603

**Published:** 2022-02-01

**Authors:** Katharine A. Leach

**Affiliations:** 1 Quality Milk Management Services, Easton Hill, UK

This is a useful study providing practical information that makes detection of subclinical mastitis more accessible to Ethiopian and Nigerian dairy farmers. I have a few suggestions:

 The clarity of reporting could be improved by making clear in each section of methods, results and discussion what is being used as the gold standard to assess the performance of the individual reagents - in some cases it is the UK commercial CMT reagent and in others it is the laboratory determined SCC.

 Traditionally, bacterial culture has been taken as the ultimate gold standard for detection of clinical mastitis (Dohoo *et al.*, 2011 ^1^), rather than counting somatic cells as suggested in paragraph 3 - this is standard for subclinical mastitis.

Methods:

 The Methods section needs a clearer explanation of the testing for SCC. It is not immediately clear whether the same samples were used for CMT/detergent tests and somatic cell counting. Was a single sample divided, part in sterile pot without preservative and part in “QMMS sample pot” (whatever that is) with preservative? This would be expected, but is not perfectly clear. The laboratory method used to determine SCC counts (Fossomatic? Coulter?...). is not mentioned at all in the methods, just that the samples were “posted within 48 hours of collection”. Please explain more.

 Dilution study section in the methods section - the first paragraph gives the impression that the objective was to evaluate the ability to detect samples with >400,000 cells/ml. However, the third paragraph states that the selection of 20 (why 20?) samples to include in this exercise was based on CMT results. I think this will be because the detergent testing needed to be done before the SCC results were available. It would be useful to explain this.

 Everything would be clearer if the word “chosen” was used in reference to the “point of significance” in the last paragraph of the methods, and, in fact, if this point was made earlier in the methods section e.g. “In this study 400,000 cells/ml, as determined by somatic cell counting (XX Method) was chosen as the point of significance, as a positive indicator of infection.”

Statistical Analysis:

 Your definition at present is not correct: “Sensitivity is the proportion of animals that test positive that truly have the disease, while specificity is the proportion of animals that test negative that indeed do not have the disease”. should read: “Sensitivity is the proportion of animals that truly have the disease that test positive, while specificity is the proportion of animals that do not have the disease that test negative.”

Results:

 I think there should be some description (e.g. boxplots), of the distribution of somatic cell counts (done by machine) for all of these results as the distribution of SCCs will have a bearing on CMT “accuracy”. The operating characteristics of a test will vary with prevalence of a disease (Bentley *et al.*, 2012 ^2^, Martin *et al.*, 1987 ^3^).

Table 1:

 Mucous membranes secrete mucus. “Mucus” is the noun and “mucous” is the adjective. You need the adjective here, the substance you have is not mucus, it merely has similar properties.

Figure 2 & Figure 3:

 Could the circles be offset? At present it appears that not all operators used all reagents, but are the circles overlapping so that an operator is sometimes obscured?

Table 4:

 Are these results *for* different reagents, *across* operators? The meaning of “range of false positives” and “range of false negatives” is not clear - I presume it is across operators. Also, to evaluate the importance of the range of false positives and false negatives we need to be given the number of samples tested in the title please.

Discussion:

 The financial information is useful and illustrates the advantage of a diluted detergent reagent. It would also be interesting if you could give an indication of the value of any milk that a producer in Nigeria or Ethiopia might be selling, at the time of the study.

 In the discussion you may also wish to comment on any differences in pathogens and (hence) chronicity between a “typical” UK case of mastitis and a “typical” African case. Stage of disease, i.e. how chronic the mastitis is at the time of sampling, will also affect the SCC and sensitivity and specificity. For example, if a large proportion of African cases are more chronic than UK cases, then sensitivity and specificity will be different when this is used in the field in Africa.

Is the work clearly and accurately presented and does it cite the current literature?

Partly

If applicable, is the statistical analysis and its interpretation appropriate?

Partly

Are all the source data underlying the results available to ensure full reproducibility?

Yes

Is the study design appropriate and is the work technically sound?

Yes

Are the conclusions drawn adequately supported by the results?

Yes

Are sufficient details of methods and analysis provided to allow replication by others?

No

Reviewer Expertise:

Dairy herd health; mastitis diagnostics

I confirm that I have read this submission and believe that I have an appropriate level of expertise to confirm that it is of an acceptable scientific standard, however I have significant reservations, as outlined above.

## References

[1] : Diagnosing intramammary infections: evaluation of definitions based on a single milk sample. *J Dairy Sci* .2011;94(1) : 10.3168/jds.2010-3559 250-61 10.3168/jds.2010-3559 21183035

[2] : Implications of the impact of prevalence on test thresholds and outcomes: lessons from tuberculosis. *BMC Res Notes* .2012;5: 10.1186/1756-0500-5-563 563 10.1186/1756-0500-5-563 23050607PMC3503683

[3] : Veterinary Epidemiology: Principles and Methods. *Iowa State University Press* .1987;1st ed.:62-73 Reference source

# Response

**Published:** 2023-02-09

**Authors:** Andrew Peters

Dear Reviewer,

 We sincerely thank you for your time and effort in providing expert assessment of our manuscript. There are constructive points raised which we have considered and hope that we have addressed suitably.

 Response to comments are listed in italics font below, with proposed additions and amendments highlighted in bold.

 This is a useful study providing practical information that makes detection of subclinical mastitis more accessible to Ethiopian and Nigerian dairy farmers. I have a few suggestions:

 1.  The clarity of reporting could be improved by making clear in each section of methods, results and discussion what is being used as the gold standard to assess the performance of the individual reagents - in some cases it is the UK commercial CMT reagent and in others it is the laboratory determined SCC.

 Traditionally, bacterial culture has been taken as the ultimate gold standard for detection of clinical mastitis (Dohoo *et al.*, 2011 1), rather than counting somatic cells as suggested in paragraph 3 - this is standard for subclinical mastitis.

*Introduction paragraph 3:*

*“SCC has been used as an indicator of mastitis in dairy herds since the 1960s (Pyörälä, 2003). Somatic cells contribute to natural defence mechanisms and include lymphocytes, macrophages, polymorphonuclear and epithelial cells (Pillai et al., 2001) and as such, these cells indicate an inflammatory process in intramammary infection. Counting somatic cells is used to distinguish between infected and uninfected quarters (Carrillo-Casas & Miranda-Morales, 2012), and has long been considered the gold standard in **subclinical** mastitis detection (Duarte et al., 2015). The California Mastitis Test (CMT) is an inexpensive, easily-applicable, cow-side test that enables the subjective assessment of the number of somatic cells present in a milk sample, as an estimation of the probability and severity of intramammary infection (Schalm & Noorlander, 1957).”*

 2.  Methods: The Methods section needs a clearer explanation of the testing for SCC. It is not immediately clear whether the same samples were used for CMT/detergent tests and somatic cell counting. Was a single sample divided, part in sterile pot without preservative and part in “QMMS sample pot” (whatever that is) with preservative? This would be expected, but is not perfectly clear. The laboratory method used to determine SCC counts (Fossomatic? Coulter?...). is not mentioned at all in the methods, just that the samples were “posted within 48 hours of collection”. Please explain more.

*Methods paragraph 2:*

*“Samples were collected between August 2020 and February 2021. All samples collected for the purpose of California Mastitis Testing were collected in sterile 50 ml **Universal containers (Teklab)** provided by Scarsdale veterinary practice, Derby, were stored at room temperature and tested within 48 hours of collection. All samples collected for **the validation study were split into aliquots for** SCC analysis and were **posted** in sterile 30 ml QMMS sample pots containing a milk preservative **(MicroTabs II, Advanced Instruments)** within 48 hours of collection. Samples were identified with numbers in chronological order of collection so as to maintain blinding of details such as sample location.”*

 3.  Dilution study section in the methods section - the first paragraph gives the impression that the objective was to evaluate the ability to detect samples with >400,000 cells/ml. However, the third paragraph states that the selection of 20 (why 20?) samples to include in this exercise was based on CMT results. I think this will be because the detergent testing needed to be done before the SCC results were available. It would be useful to explain this.

*Dilution study paragraph 3:*

*“The five dilutions for each of the six detergents were then tested with 20 quarter milk samples **; 20 samples provided clear indication of the most effective dilutions required to indicate subclinical mastitis. **All samples were screened for inclusion in the study using the commercial UK CMT to ensure 15 samples with known high SCC values (CMT score 2–3) were included as well as five samples with known low SCC (CMT score 0–1) to test for false positives. Scores were based on reaction descriptions shown in Table 1.”*

 4.  Everything would be clearer if the word “chosen” was used in reference to the “point of significance” in the last paragraph of the methods, and, in fact, if this point was made earlier in the methods section e.g. “In this study 400,000 cells/ml, as determined by somatic cell counting (XX Method) was chosen as the point of significance, as a positive indicator of infection.”

*Validation study paragraph 3:*

*“The study identifies the point of significance as 400,000 cells per ml, determined by the laboratory SCC ( **Fossomatic method, Delta CombiScope – Model FTIR 400, Netherlands), according to the FIL.IDF 148 A: 95 norm**. Samples with SCC >400,000 were **chosen** **as the point of significance**, as a positive indicator of **infection** and thus we can expect a CMT score of 2–3, and samples <400,000 were deemed as weakly positive to negative, and thus we can expect a score of 0–1 on the CMT.*

 5.  Statistical Analysis:  Your definition at present is not correct: “Sensitivity is the proportion of animals that test positive that truly have the disease, while specificity is the proportion of animals that test negative that indeed do not have the disease”. should read: “Sensitivity is the proportion of animals that truly have the disease that test positive, while specificity is the proportion of animals that do not have the disease that test negative.”

*“Validity of the CMT results using different detergents was assessed by comparing test sensitivity and specificity. **Sensitivity is the proportion of animals that truly have the disease that test positive, while specificity is the proportion of animals that do not have the disease that test negative**.  The results of each CMT were validated against a SCC, and sensitivity and specificity were calculated using the formula in Table 2 ( Thrusfield, 2018b). High sensitivity minimises the number of false-negative results, while high specificity minimises the number of false-positive results. Confidence intervals were calculated using Wilson’s method ( Thrusfield, 2018a).”*

 6.  Results:  I think there should be some description (e.g. boxplots), of the distribution of somatic cell counts (done by machine) for all of these results as the distribution of SCCs will have a bearing on CMT “accuracy”. The operating characteristics of a test will vary with prevalence of a disease (Bentley *et al.*, 2012 2, Martin *et al.*, 1987 3).

*Whilst we acknowledge that there is variability in SCC, assessing the accuracy of the CMT was beyond the scope of the project.*

*Discussion seventh paragraph:*

*“The CMT is subjective in nature, which allows room for bias as well as operator error. A laboratory SCC automatically counts cells within a sample, reducing operator error and removing bias, but it is not without an error margin, **assessment of** **which was beyond the scope of this study**. The prolonged retention of samples, postal disturbances, transport and delivery may all contribute to sample modification. Additionally, the SCC also does not take away the value of a clinical exam, as mastitis and other mammary disease may not, in the rare occasion, be reflected in high SCC. All samples in this study are taken from UK Holstein Friesians cows. This study, therefore, assumes that indigenous breeds native to Ethiopia and Nigeria, though managed differently, will respond similarly to the CMT.”*

 7.  Table 1:  Mucous membranes secrete mucus. “Mucus” is the noun and “mucous” is the adjective. You need the adjective here, the substance you have is not mucus, it merely has similar properties.

**Category                 Score     Description of Reaction**

 Negative                  0 ****Mixture of milk and test fluid remains the same and can easily be shaken.

 Weakly Positive       1 ****Mixture has a slight **mucous** appearance but can easily be shaken.

 Positive                    2 ****Unmistakable **mucous** formation can be seen, it is possible still to tip a                                                     small proportion of the mixture out.

 Strongly Positive      3 ****Jelly-like consistency is formed and it is difficult to shake the mixture. It is                                                   no longer possible to tip out any surplus liquid.

 8.  Figure 2 & Figure 3: Could the circles be offset? At present it appears that not all operators used all reagents, but are the circles overlapping so that an operator is sometimes obscured?

*Data points have been offset (cannot include figures here).*

 9.  Table 4: Are these results *for* different reagents, *across* operators? The meaning of “range of false positives” and “range of false negatives” is not clear - I presume it is across operators. Also, to evaluate the importance of the range of false positives and false negatives we need to be given the number of samples tested in the title please.

*Table 4. The average sensitivity, specificity, positive predictive value (PPV) and negative predictive value (NPV) of using the CMT to determine high SCC (>400,000 cells per ml) **for **different domestic-based CMT reagents **across** operators **. n = 132***

 10. Discussion: The financial information is useful and illustrates the advantage of a diluted detergent reagent. It would also be interesting if you could give an indication of the value of any milk that a producer in Nigeria or Ethiopia might be selling, at the time of the study.

*Discussion paragraph 5:*

*“Availability and cost are both important factors in being able to purchase reagents. In selecting detergents for the study, national availability was a selection criterion, along with detergents being in the higher end of the market, as better-quality products had been found to be more effective by Leach et al. (2008). For the CMT, the required volume of test fluid per cow is 12ml (3 ml per quarter); one litre of test fluid would therefore be sufficient for 83 cows. At the time of writing, the recommended Ethiopian detergent ‘Shagan’ costs 92–138 Ethiopian Birr (ETB) (equivalent to 2.0–3.1 USD) for one litre. To make one litre of the CMT fluid at the recommended dilution, 275 ml of Shagan is required, equivalent to 25–38 ETB (0.6–0.8 USD), equating to 0.1 ETB per quarter (0.002 USD). The recommended Nigerian detergent ‘Sunlight’ costs 800–866 Nigerian Naira (NGN) (equivalent to 1.9-2.1 USD) for one litre. To make one litre of CMT fluid at the recommended dilution, 350 ml of Sunlight is required, equivalent to 280–303 NGN (0.6-0.7 USD). This equates to around 0.9 NGN per quarter (0.002 USD). Commercial reagent for the UK CMT costs between 6.9–8.3 USD for one litre. The recommended local detergents, therefore, cost around one tenth of the commercial product and are both readily available. **To give some context, at the time of the study the retail price for a litre of milk in Ethiopia ranges from 50-60 ETB (0.9-1.2 USD) and the price for a smallholder selling locally on the informal market is between 30-35 ETB (0.6-0.7 USD) per litre.  In Nigeria, these prices range from 250-300 NGN (0.6-0.7 USD) for retail and 150 NGN (0.4 USD) per litre for a smallholder during the rainy season and 250 NGN (0.6 USD) per litre during the dry season.**”*

 11.  In the discussion you may also wish to comment on any differences in pathogens and (hence) chronicity between a “typical” UK case of mastitis and a “typical” African case. Stage of disease, i.e. how chronic the mastitis is at the time of sampling, will also affect the SCC and sensitivity and specificity. For example, if a large proportion of African cases are more chronic than UK cases, then sensitivity and specificity will be different when this is used in the field in Africa.

*Discussion paragraph seven:*

*“The CMT is subjective in nature, which allows room for bias as well as operator error. A laboratory SCC automatically counts cells within a sample, reducing operator error and removing bias, but it is not without an error margin. The prolonged retention of samples, postal disturbances, transport and delivery may all contribute to sample modification. Additionally, the SCC also does not take away the value of a clinical exam, as mastitis and other mammary disease may not, in the rare occasion, be reflected in high SCC. All samples in this study are taken from UK Holstein Friesians cows. This study, therefore, assumes that indigenous breeds native to Ethiopia and Nigeria, though managed differently, will respond similarly to the CMT. **Pathogens are thought to be similar in African cases, but the availability and types of treatment may be different. Low milk production, how chronic the mastitis is at time of sampling and nutritional status of cows may all effect the sensitivity and specificity when used in the field.”***
